# mHealth technology identifies visual impairment in young children

**DOI:** 10.1002/ctm2.1238

**Published:** 2023-04-11

**Authors:** Wenben Chen, Yuanfan Lin, Ruiyang Li, Andi Xu, Yonghao Deng, Haotian Lin

**Affiliations:** ^1^ State Key Laboratory of Ophthalmology, Zhongshan Ophthalmic Center Sun Yat‐sen University Guangdong Provincial Key Laboratory of Ophthalmology and Vision Science, Guangdong Provincial Clinical Research Center for Ocular Diseases Guangzhou China; ^2^ Zhongshan School of Medicine Sun Yat‐sen University Guangzhou China; ^3^ Hainan Eye Hospital and Key Laboratory of Ophthalmology Zhongshan Ophthalmic Center, Sun Yat‐sen University Haikou China; ^4^ Center for Precision Medicine and Department of Genetics and Biomedical Informatics Zhongshan School of Medicine, Sun Yat‐sen University Guangzhou China

Visual impairment is a widespread public health issue that can have a negative impact on quality of life, education and socioeconomic development.^1^ The early stages of life, particularly early childhood (infancy and toddlerhood), are crucial periods for visual development, during which detection and treatment of ocular pathology can prevent irreversible vision loss.^2^, ^3^ Unfortunately, young children are often unable to complain of ocular symptoms or unwilling to participate in standard vision tests, making it challenging to assess their visual functions. Currently, professional pediatric ophthalmologists are needed to evaluate the vision function of young children by observing their reaction to visual stimuli,^4^ but these tests have limited repeatability in large‐scale screening studies, which hinders their broad application, especially in regions with limited medical resources.^5^ Therefore, it is crucial to develop an effective and user‐friendly tool for the early detection of visual impairment in young children.

## 1 SEE THE LIGHT

Ocular abnormalities often manifest with typical phenotypic features, such as leukocoria^6^ or eyelid drooping.^7^ In our previous study, we found that dynamic aberrant behavioral features, such as abnormal ocular movement and fixation patterns, were significantly related to visual impairment of young children.^8^ Although these phenotypic features can be useful clues in ophthalmic practice for diagnosing visual impairment, there is still a lack of valid and practical detection tools that can systematically record, analyse, and apply these features in real‐world settings. Advancements in mobile health (mHealth) and artificial intelligence technology in digital medicine have created an excellent opportunity to record and analyse phenotypic features for early identification of visual impairment in young children. However, developing a mHealth system for large‐scale applications presents challenges. The first is the high requirements for the stability of the system due to the difficulty to collect qualified phenotypic data of young children in chaotic environments with interference or noise. Additionally, it is difficult to generalize the system for large‐scale applications and to provide rigorous evidence of its feasibility in clinical application. These challenges highlight the current lack of such a practical tool for early detection of visual impairment in young children.

## 2 APOLLO INFANT SIGHT

**FIGURE 1:**
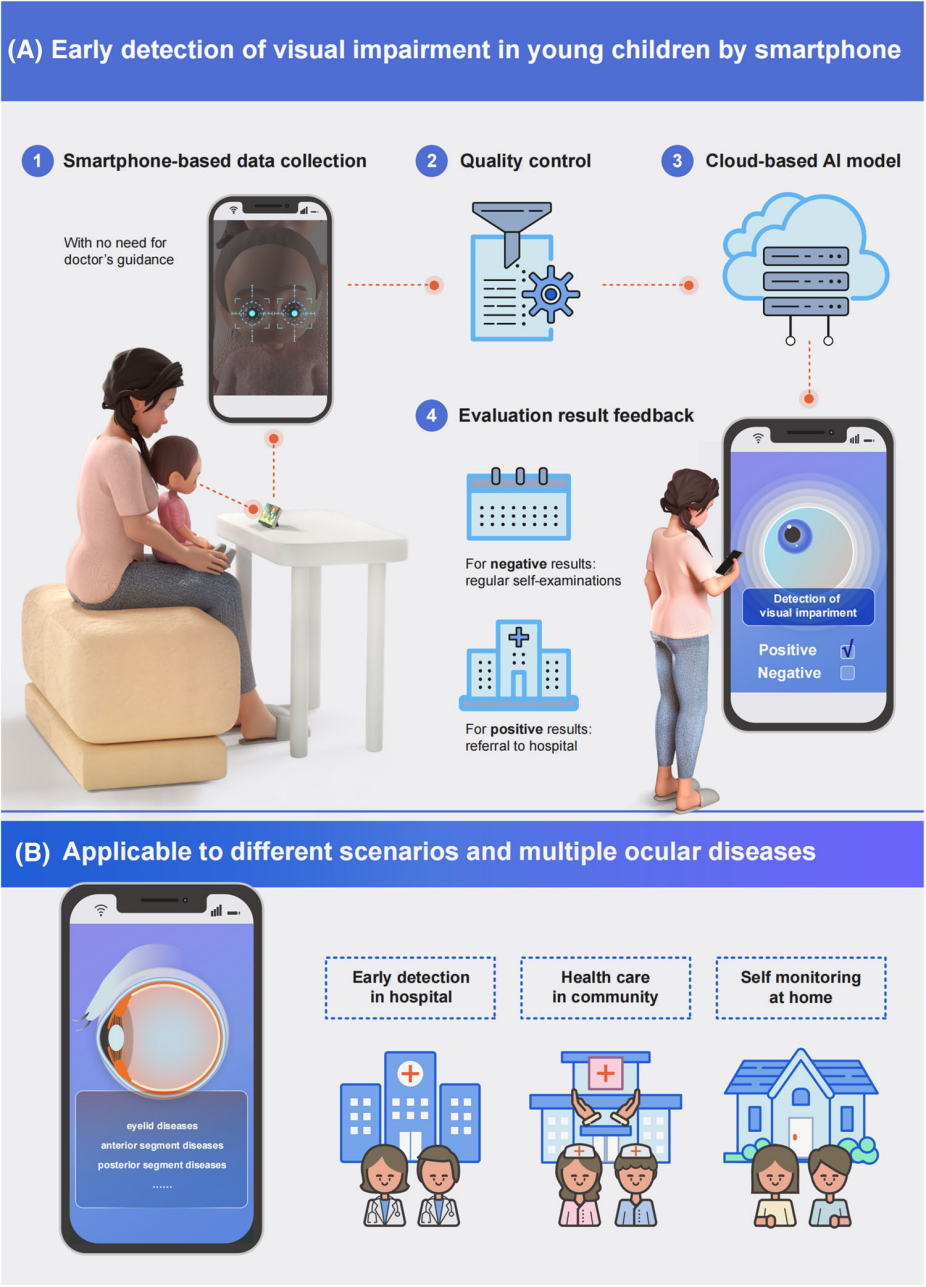
(A) The process of using the AIS system based on smartphone to identify visual impairment of young children. (B) The application scenarios and eye diseases of the AIS system. AIS, Apollo Infant Sight.

We have created a mHealth system called the Apollo Infant Sight (AIS), which includes an app collecting data and a deep learning (DL) system analyzing the collected data.^9^ The DL model was trained with 25 972 800 frames of video from 3652 children to detect visual impairment caused by 16 common eye diseases in children aged ≤48 months. The app guides healthcare professionals, volunteers, parents and caregivers to record videos of children's gaze patterns while watching a cartoon video to ensure steady gaze. The recorded videos are analysed by the DL system, which returns the results to the app (Figure 1A). To eliminate environmental interference and ensure the reliability of the system in the real world, we also designed a quality control module in our model, including face detection, facial key point localization and other quality‐checking methods.

We evaluated the performance of our automated intelligent detection system (AIS) in both clinical and at‐home settings (Figure 1B). Initially, we developed and thoroughly validated the system at the Zhongshan Ophthalmic Center, where it achieved an area under the receiver operating characteristic curve (AUROC) of .940. Subsequently, we conducted a multicenter study at four other hospitals in various provinces in China to assess the system's generalizability in different clinical settings, resulting in an AUROC of .843. For at‐home settings, we recruited untrained parents or caregivers from the Guangdong area online to evaluate the system via their smartphones in community settings. Our system achieved an AUROC of .859, demonstrating its versatility under various testing conditions. Our study indicates that AIS is a promising tool for early detection of visual impairment in young children, enabling timely referral and prompt intervention to improve visual outcomes.

## 3 FUTURE OUTLOOK

We have comprehensively assessed AIS by internal validation, external validation and at‐home implementation, preliminarily verifying the feasibility of using the system for early detection of visual impairment in young children. In the future, a large‐scale screening trial to verify the utility of AIS in population‐based screening remains to be conducted. Additionally, further work should improve the performance of AIS for reducing the missed diagnosis of patients with slight visual impairment and reduce the risk of privacy exposure by using ‘digital mask’, a technology based on 3D reconstruction and DL algorithms to erase identifiable features irreversibly and retain disease‐relevant features of facial images.^10^ Besides, applying AIS to detect visual impairment caused by some systemic and neurological diseases would be of great clinical significance and its feasibility needs to be further validated.

## CONFLICT OF INTEREST STATEMENT

The authors declare no conflicts of interest.
